# Saikosaponin D Is Associated with Anti-Tumor Effects and Markers of Autophagy and Endoplasmic Reticulum Stress in Human Endometrial Cancer Ishikawa Cells

**DOI:** 10.3390/nu18081221

**Published:** 2026-04-13

**Authors:** Xiu-Xiu Zhang, Tong-Tong Tang, Xiao-Mei Ma, Kiran Thakur, Fei Hu, Jian-Guo Zhang, Yi-Long Ma, Zhao-Jun Wei

**Affiliations:** 1 School of Food Science and Biological Engineering, Hefei University of Technology, Hefei 230009, Chinahufei@hfut.edu.cn (F.H.); zhangjianguo@hfut.edu.cn (J.-G.Z.); yilong.ma@hfut.edu.cn (Y.-L.M.); 2 Ningxia Key Laboratory of Development and Utilization of Specialty Food Resources, School of Biological Science and Engineering, North Minzu University, Yinchuan 750021, China; 2025910@nmu.edu.cn

**Keywords:** Saikosaponin D, immunohistochemistry, autophagy, endoplasmic reticulum stress, Ishikawa

## Abstract

**Background/Objectives:** Saikosaponin D (SSD) is a bioactive compound from traditional Chinese herbs with known anti-tumor activities, including apoptosis induction, autophagy modulation, and inhibition of cell migration and invasion. However, the mechanisms underlying its effects on human endometrial cancer Ishikawa cells remain elusive. This study aimed to investigate the anti-tumor effects of SSD on EC Ishikawa cells and elucidate the molecular pathways involved, focusing on DNA damage, cell cycle regulation, autophagy, endoplasmic reticulum (ER) stress, and AMPK signaling. **Methods:** We performed in vitro experiments using Ishikawa cells and in vivo studies using a female BALB/c nude mouse xenograft model. DNA damage was assessed via comet assay, intracellular Ca^2+^ concentration via Fluo-3 AM staining, autophagy via transmission electron microscopy, and apoptosis via flow cytometry. Autophagy was inhibited using 3-methyladenine, and ER stress was modulated with the PERK inhibitor GSK2656157. Protein expression levels of related genes were analyzed by western blotting. No preregistration number or CONSORT details applied, as this was a pre-clinical study. **Results**: SSD treatment was associated with DNA damage and G2/M phase cell cycle arrest in Ishikawa cells both in vitro and in vivo. SSD was associated with an increased LC3II/LC3I ratio and activation of the AMPK pathway. It was also associated with ER stress, as evidenced by downregulation of PERK, mTOR, and eIF2α, and upregulation of p-eIF2α. Furthermore, SSD was associated with modulation of the AMPK signaling pathway to inhibit cell migration and invasion. **Conclusions:** SSD exerts anti-tumor effects on human EC Ishikawa cells in vitro and in vivo through mechanisms involving DNA damage, G2/M arrest, autophagy, ER stress, and AMPK-mediated inhibition of migration and invasion. These findings suggest that SSD may represent a potential therapeutic agent for EC.

## 1. Introduction

Endometrial cancer (EC) is one of the most common malignant tumors in women, and its incidence and mortality rates are gradually increasing worldwide [1]. Statistically, the global figure for EC is likely to exceed 400,000 new cases, with a mortality rate of nearly 100,000 cases [1]. It is worth noting that the average age of incidence of EC is approximately 63 years, but in recent years, there has been a dramatic increase in female patients under 50 years of age. Current standard treatments, including surgery, chemotherapy, and radiotherapy, are associated with severe side effects such as cardiotoxicity, neurotoxicity, and endocrine disruption, significantly compromising patients’ quality of life [2]. Furthermore, a substantial proportion of EC patients develop resistance to conventional chemotherapeutic agents, leading to tumor recurrence and poor prognosis [3]. These limitations create an urgent need for alternative therapeutic strategies that offer improved efficacy with reduced toxicity.

Natural phytochemicals have emerged as promising candidates for cancer therapy due to their multi-target mechanisms, lower toxicity profiles, and potential to overcome drug resistance [4,5]. Programmed cell death encompasses multiple forms, including apoptosis, autophagy, ferroptosis, necroptosis, and pyroptosis [6,7]. Among these, autophagy represents a distinct mode of programmed cell death, and endoplasmic reticulum (ER) stress serves as a critical upstream event capable of activating various cell death pathways, both of which are particularly relevant to the mechanism of action of SSD, as detailed below. These cell death modalities are intricately linked to fundamental cellular signaling pathways. Numerous anti-tumor natural phytochemicals have been shown to modulate these pathways by regulating the expression of key receptors, proteins, and genes, thereby inducing programmed cell death in cancer cells [8].

Both cell cycle and endoplasmic reticulum (ER) stress are strongly linked to apoptosis. DNA damage occurs when genomic DNA is attacked by both external and internal sources. DNA-damaged cells activate cell cycle checkpoints, leading to cell cycle arrest and, if not repaired in time, apoptosis [9]. It has been shown that co-treatment of Cal-33 cells with resveratrol and quercetin resulted in the appearance of comet tails and S-phase cell cycle arrest [10]. Ginkgo biloba can induce apoptosis by blocking the cycle through the regulation of p21 and Cyclin A [11]. ER stress represents a particularly attractive therapeutic target in EC. Endometrial tumors exhibit high basal ER stress levels due to their rapid proliferation rates and elevated protein synthesis demands [12,13]. Accumulation of misfolded proteins within the ER lumen leads to calcium dysregulation and activation of the unfolded protein response (UPR), which can trigger apoptosis when prolonged or severe [14]. Proteins that are misfolded within the endoplasmic reticulum are grouped together within the lumen, leading to an increase in Ca^2+^, which is detrimental to cell function and survival [15]. It has been shown that misfolded proteins in the endoplasmic reticulum trigger the UPR through activation of stress sensors such as IRE1α and PERK, with downstream effectors including ATF4, ultimately leading to apoptosis under persistent stress [16]. Naringin induces a ROS-mediated stress response in the endoplasmic reticulum, leading to apoptosis via the PERK/eIF2α/ATF4/CHOP pathway [17].

Autophagy is a basic survival mechanism by which cells protect themselves or maintain normal cellular activities by degrading or recycling intracellular nutrients [18,19]. As a modulator of many cancer genes and tumor suppressor genes [20], it is known that autophagy is engaged in promoting oncogenesis as well as cancer progression and suppression. Curcumin was reported to induce autophagy, as evidenced by LC3-II conversion, Beclin-1 accumulation, p62/SQSTM1 degradation, and increased acidic vesicular organelle (AVO) formation [21]. In another study, periplocin could induce autophagy by activating the AMPK/mTOR pathway in pancreatic cancer cells [22].

Saikosaponin D (SSD), extracted from the traditional Chinese medicinal herb *Bupleurum chinense* (Chaihu), represents a particularly promising natural anti-tumor agent. SSD exhibits diverse pharmacological activities, including anti-tumor, hypolipidemic, and antidepressant effects [23], with a demonstrated safety profile and broad prospects for pharmaceutical applications. The Ishikawa cell line serves as an excellent model for investigating EC biology, as it retains hormone responsiveness and represents the endometrioid subtype, the most common form of EC [24]. In our previous study [25], we established that SSD effectively inhibits proliferation and cell migration and invasion in Ishikawa cells, providing a solid foundation for the current mechanistic investigation. To this end, the present study employs both in vitro (Ishikawa cells) and in vivo (xenograft mouse model) systems to explore potential associations between SSD exposure and anti-tumor phenotypes in EC, specifically investigating correlations with DNA damage, cell cycle distribution, ER stress responses, autophagy markers, and changes in metastatic potential. By exploring these interconnected cellular processes and their underlying molecular pathways, we seek to generate hypotheses regarding SSD as a potential therapeutic candidate for endometrial cancer treatment, offering a natural alternative with reduced toxicity compared to conventional therapies.

## 2. Materials and Methods

### 2.1. Chemicals and Reagents

Saikosaponin D (purity > 97%) was purchased from Must Biotech Co., Ltd. (Chengdu, China). RPMI-1640 medium and fetal bovine serum were obtained from Jiangsu Kaiji Biotech Co., Ltd. (Nanjing, China) and Suzhou Shuangru Biotech Co., Ltd. (Suzhou, China), respectively. Primary antibodies were purchased from Cell Signaling Technology (Danvers, MA, USA). 3-methyladenine (3-Ma) and GSK2656157 were obtained from MedChem Express (Monmouth Junction, NJ, Hoboken,USA). The Comet Electrophoresis Assay Kit, Ca^2+^ Detection Kit, Apoptosis Detection Kit, BCA protein assay kit, SDS-PAGE gel preparation kit, and ECL Western blotting substrate were purchased from Jiangsu Kaiji Biotech Co., Ltd. (Nanjing, China), Bestbio Biotech Co., Ltd. (Shanghai, China), and Tanon Life Science Co., Ltd. (Shanghai, China), respectively.

### 2.2. Cell Culture

Frozen endometrial cancer Ishikawa cells were resuscitated and cultured in culture dishes containing RPMI-1640 culture medium (KGL1501-500) and fetal bovine serum (A511-001). The cells were placed in an incubator containing 5% CO_2_ and 95% air at a constant temperature of 37 °C under sterile culture conditions [25], and the revived cells were used for subsequent experiments.

### 2.3. In Vivo Tumor Xenograft Model in Nude Mice

All the protocols of in vivo animal studies complied with the Hefei University of Technology’s guidance and animal care protocols in compliance with national and international legal policies (Animal Ethics Approval Number: HFUT20240114002; 14 January 2024). Cultured Ishikawa suspension cells were injected subcutaneously from the right side into the axilla of each female BALB/c nude mouse (4–5 weeks old) at a density of 1 × 10^7^/0.1 mL to establish the nude mouse tumor xenograft model. The diameter of the transplanted tumor was measured with a vernier caliper every two days by an investigator blinded to group allocation, and when the tumor grew to 80–100 mm^3^ (about 15 days), it indicated that the nude mouse tumor xenograft model was successfully established. A total of 16 mice were used in the experiment. Animals were stratified by body weight and randomly allocated to two groups (*n* = 8 per group): the SSD treatment group and the model group. SSD (5 mg/kg) was dissolved in saline containing 0.5% Tween-80 and 5% DMSO and administered intraperitoneally to the treated group of nude mice. The control group received an equal volume of vehicles (saline containing 0.5% Tween-80 and 5% DMSO) every day, and the body weights of the nude mice and the volume of the tumors were recorded every two days. Finally, after 21 days of administration, the nude mice used in the experiment were sacrificed, and the tumor tissues, heart, spleen, lungs, liver, and kidneys were partly stored in fixative and partly embedded in paraffin wax for further analysis [26]. The tumor characteristics were calculated using the following equations:

(1) $$Tumor\;volume\;(TV)=\frac{{\mathrm{ab}}^{2}}{2}$$

where a and b denote length and width respectively.

(2) $$Relative\;tumor\;volume\;(RTV)=\frac{V_{t}}{V_{0}}$$

V_0_ is the tumor volume measured at the time of randomization; V_t_ is the tumor volume at each measurement.

(3) $$Relative\;tumor\;proliferation\;rate\;T/C\;(\%)=\frac{TRTV}{CRTV}\times 100\%$$

TRTV: RTV in treatment group; CRTV: RTV in untreated group.

### 2.4. H&E Staining Assay

After 21 days of SSD (5 mg/kg) administration to nude mice, all the mice were sacrificed, and the tumor tissues, heart, liver, spleen, lungs, kidneys and other organs were extracted for fixation, and then the paraffin-embedded tissues and organs were put into an embedding machine. Firstly, the wax was melted and put into an embedding frame, and then the tissues that needed to be embedded were taken out from the tissue processor, transferred into the embedding frame and marked according to the requirements of the embedding surface, and then cooled at −20 °C and allowed to solidify. The embedded tissues and organs were taken out and trimmed, cut into 4–5 μm thick sections with a microtome, flattened in warm water, and baked in an oven at 60 °C. The paraffin sections were soaked in xylene twice, then sequentially in a gradient ethanol solution, and then washed in PBS. After dewaxing and rehydration, the sections were stained according to the hematoxylin-eosin (H&E) staining method, and the stained sections were dried and mounted. Finally, the sections were observed and photographed under bright-field conditions using a fluorescence microscope [27].

For quantitative histomorphometric analysis of tumor necrosis, three representative H&E-stained sections per tumour (taken at 100 μm intervals from the central region of each tumor) were analyzed by an investigator blinded to group allocation. The total tumor cross-sectional area and the central necrotic zone area were measured using ImageJ (version 2.1.4.7, Bethesda, MA, USA). Necrotic zones were identified by characteristic features, including loss of cellular architecture, eosinophilic (pink) cytoplasmic staining, nuclear fragmentation or loss (karyorrhexis/karyolysis), and presence of inflammatory cell infiltration. The necrotic area percentage was calculated as: (necrotic zone area/total tumor cross-sectional area) × 100%. Measurements from three sections per tumor were averaged to obtain a single value per animal. Data are expressed as mean ± standard deviation (*n* = 8 per group).

### 2.5. Combinatorial Comet Test and Single-Cell Gel Assay

Single-cell gel electrophoresis (comet assay) is a preferred approach to measure DNA damage and repair. Ishikawa cells were collected after SSD (16 μM) treatment for 24 h and resuspended in PBS to reach a density of 1 × 10^6^ cells/mL. Then, subsequently, the first layer of gel was constructed by selecting normal melting point agarose (NMA) solution and spreading it evenly on a frosted slide. After solidification, the coverslip was removed, and 75 μL of 0.7% low melting point agarose (LMA) solution and 10 μL of cell suspension were mixed well and pipetted onto the first layer of gel. Another 75 μL of 0.7% LMA solution and 10 μL of cell suspension were mixed well and pipetted onto the first layer of gel, covered with a new coverslip and placed at 4 °C to solidify [28]. The coverslip was removed, and another layer of gel was constructed by adding another drop of 0.7% LMA solution. After solidification, the coverslip was removed, and the slides were placed on a flat tray, followed by the addition of pre-cooled lysis buffer solution onto the slides. After 2 h, the slides were removed and rinsed with PBS. After that, the slides were placed in a horizontal electrophoresis tank, and the newly prepared alkaline electrophoresis buffer was poured in to carry out the horizontal electrophoresis experiments at a voltage of 25 V. After the electrophoresis, 0.4 mM Tris-HCl (pH 7.5) buffer was added, which was used for neutralization, and then propidium iodide (PI) was added to carry out the staining, and finally, the DNA damage (comet tails) was observed and photographed and analyzed under the fluorescence microscope [29].

### 2.6. Detection for Ca^2+^ Concentration

Fluo-3 AM is among the most used probes that detect intracellular calcium ion levels. A total of 1.8 × 10^5^ cells/mL of Ishikawa cells were selected and seeded in cell culture dishes. After 16 h, the cells adhered, and different concentrations of SSD were added (15, 16, and 17 μM). The cells were collected into a cell suspension after 24 h of the SSD treatment. After that, the cells were incubated for 60 min with 0.5–5 μM of Fluo-3 AM at 37 °C for 60 min to ensure the conversion of the probe from Fluo-3 AM to Fluo-3 in the cells. After that, cells were analyzed using a flow cytometer [30].

### 2.7. Transmission Electron Microscopy

Cells with a density of 1.8 × 10^5^ cells/mL were seeded in cell culture dishes for 16 h and allowed to adhere during the logarithmic growth phase. Following SSD (16 μM) treatment for 24 h, the supernatant was removed, then the adherent cells were scraped off with a cell spatula, washed with PBS, centrifuged, and added to electron microscopy fixative and placed at 4 °C for overnight fixation. The fixed cells were embedded in 1.0% agarose gel, washed with 0.1 M PBS, and then fixed in 1% osmium tetroxide in 0.1 M PBS (pH 7.4) at room temperature for 2 h. Subsequently, the cells were dehydrated in ethanol and acetone, then infiltrated and embedded with embedding agent, and the samples were then cut into 60–80 nm ultra-thin sections and stained with uranyl acetic acid and lead citrate. The sections were further dried overnight at room temperature prior to observation and image acquisition under a transmission electron microscope (TEM) [16].

### 2.8. Detection of Apoptosis in 3-Ma-Treated Cells Combined with SSD

To investigate the functional role of SSD-induced autophagy in apoptosis, Ishikawa cells were pretreated with the autophagy inhibitor 3-methyladenine (3-Ma, 1 mM, Sigma-Aldrich, St. Louis, MI, USA) for 2 h, followed by SSD (16 μM) treatment for 24 h. Cells were divided into four groups: control, 3-Ma alone, SSD alone, and 3-Ma + SSD. After treatment, both floating and adherent cells were collected, washed with PBS, and stained with Annexin V-FITC/PI using the Annexin V Binding Buffer according to the manufacturer’s instructions. Stained cells were analyzed by flow cytometry [25].

### 2.9. Immunohistochemistry of Tumor Tissues

Tissue sections from nude mice were deparaffinized and rehydrated in xylene, gradient ethanol and PBS, after which the tissue sections were placed in EDTA (pH 6.0) antigen retrieval buffer, and then placed in a microwave oven for antigen retrieval. After cooling at room temperature, a few drops of 3% H_2_O_2_-methanol solution were added to each section to inhibit the endogenous peroxidase activity, and then the sections were blocked with goat-derived serum at room temperature. The sections were incubated with the blocking serum at room temperature for 1 h, gently blotted to remove the excess solution, and then washed with antibodies against Caspase3, MMP2, MMP9, and LC3B at 4 °C overnight, and then washed with PBST three times after the incubation, and then further incubated with secondary antibody for 30 min at 37 °C for DAB staining, followed by counterstaining with hematoxylin and washed with distilled water. Finally, protein expression was analyzed and photographed under a light microscope [31].

### 2.10. Treatment with PERK Inhibitor GSK2656157

GSK2656157 is an inhibitor of the PERK, a protein associated with the ER stress pathway. Cells were seeded into a cell culture dish at 1.8 × 10^5^ cells/mL, and the medium was discarded after 16 h [32]. Cells were randomly allocated to four treatment groups: (1) combined GSK2656157 (1 μM) plus SSD (16 μM); (2) SSD (16 μM) alone; (3) GSK2656157 (1 μM) alone; and (4) untreated control.

### 2.11. Western Blotting

Western blotting involves transferring protein to the PVDF membrane, and then the principle of specific binding of antigen–antibody is applied. Total protein was extracted using RIPA lysis buffer (Beyotime Biotechnology, P0013B, Shanghai, China) containing 1% PMSF (phenylmethylsulfonyl fluoride) and 1% phosphatase inhibitor cocktail. Protein concentration was determined using the BCA protein assay kit (Jiangsu Kaiji Biotech Co., Ltd., Nanjing, China). Thirty micrograms (30 μg) of protein per sample were loaded onto SDS-PAGE gels. The primary antibodies used were as follows: LC3B (Cell Signaling Technology, #3868, 1:1000), p62/SQSTM1 (Cell Signaling Technology, #8025, 1:1000, Danvers, MA, USA), PERK (Cell Signaling Technology, #3192, 1:1000, Danvers, MA, USA), p-eIF2α (Cell Signaling Technology, #3398, 1:1000, Danvers, MA, USA), eIF2α (Cell Signaling Technology, #5324, 1:1000, Danvers, MA, USA), mTOR (Cell Signaling Technology, #2983, 1:1000, Danvers, MA, USA), p-mTOR (Cell Signaling Technology, #5536, 1:1000, Danvers, MA, USA), AMPK (Cell Signaling Technology, #5831, 1:1000, Danvers, MA, USA), p-AMPK (Cell Signaling Technology, #2535, 1:1000, Danvers, MA, USA), and β-actin (Cell Signaling Technology, #4970, 1:2000, Danvers, MA, USA). After overnight incubation with primary antibodies at 4 °C, membranes were washed and incubated with HRP-conjugated secondary antibodies (Cell Signaling Technology, #7074 or #7076, 1:2000, Danvers, MA, USA) for 1 h at room temperature. Protein bands were visualized using ECL Western blotting substrate and detected in a gel imaging system [16].

### 2.12. Statistical Analysis

All data from three or more replicated experiments were statistically analyzed by Origin 2021 (version 2024 (10.1)) and SPSS software (version 26) and expressed as mean ± standard deviation (*n* ≥ 3). Student’s *t*-test was used for comparisons between two groups for single timepoint measurements. One-way ANOVA followed by Tukey’s post hoc test was used for multiple group comparisons. For longitudinal in vivo measurements (tumor volume and body weight), which were recorded every two days over the 21-day experimental period, two-way repeated measures ANOVA was employed, with time as the within-subjects factor and treatment group (Control vs. SSD) as the between-subjects factor. This analysis appropriately accounts for the correlation between repeated measurements from the same animal. Mauchly’s test of sphericity was performed to assess the sphericity assumption; when violated, the Greenhouse-Geisser correction was applied to adjust the degrees of freedom. When significant main effects of treatment, time, or treatment × time interactions were detected, Bonferroni-corrected pairwise comparisons were conducted to identify specific time points showing significant between-group differences, while controlling for family-wise error rate across multiple comparisons. Significant differences between comparison groups were detected at *p* < 0.05.

## 3. Results

### 3.1. Association of SSD Treatment with Inhibitory Effects on Ishikawa Cells In Vitro and In Vivo

**Figure 1:**
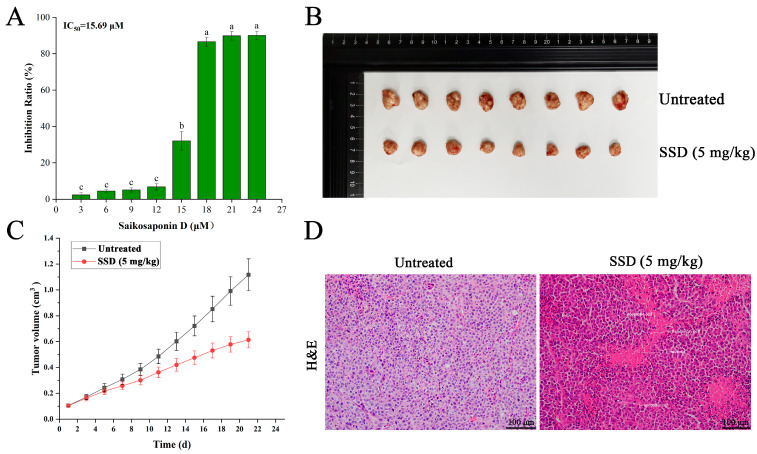
Association of SSD treatment with inhibitory effects on human endometrial cancer Ishikawa cells in vitro and in vivo. (**A**) IC_50_ values of SSD on human endometrial cancer cells after 24 h of exposure. Data represent mean ± standard deviation from three independent biological replicates (*n* = 3). (**B**) comparison of tumor tissue morphology (*n* = 8 per group). (**C**) In vivo tumor growth assessment in nude mice (*n* = 8 per group; two-way repeated measures ANOVA: treatment effect F(1,14) = 12.17, *p* < 0.001; time effect F(10,140) = 4436.04, *p* < 0.001; treatment × time interaction F(10,140) = 534.83, *p* < 0.001) and; (**D**) representative H&E staining of tumor sections (*n* = 8 per group). Lowercase letters indicate significant differences among the groups (*p* < 0.05).

**Figure 2:**
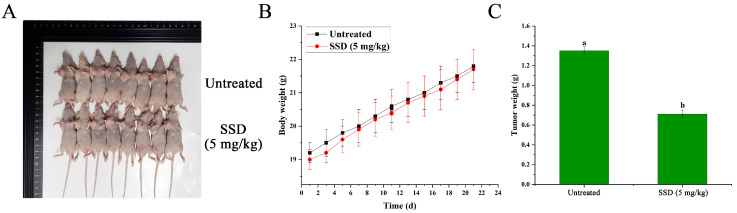
Effect of SSD on nude mice with hormonal tumors. (**A**) Morphological size of nude mice with Ishikawa cell xenografts sacrificed after 21 days of treatment (*n* = 8 per group); (**B**) difference in body weight of mice after 21 days of treatment (*n* = 8 per group; two-way repeated measures ANOVA: treatment effect F(1,14) = 0.58, *p* = 0.449; treatment × time interaction F(10,140) = 1.32, *p* = 0.221); (**C**) difference in tumor tissue weight of mice after 21 days of treatment (*n* = 8 per group, Student’s *t*-test, *p* < 0.05). Lowercase letters indicate significant differences among the groups (*p* < 0.05).

According to our previous findings [25], SSD was associated with a significant inhibitory effect on human endometrial cancer Ishikawa cells in vitro (Figure 1A). To investigate potential associations between SSD exposure and anti-tumor phenotypes in human endometrial cancer Ishikawa cells, we established an in vivo tumor xenograft model by injecting Ishikawa cells into the axillae of BALB/c female nude mice. Longitudinal analysis of tumor growth using two-way repeated measures ANOVA revealed significant differences between treatment groups over the 21-day observation period. The analysis demonstrated a significant main effect of treatment [F(1,14) = 12.17, *p* < 0.001], a significant main effect of time [F(10,140) = 4436.04, *p* < 0.001], and a significant treatment × time interaction [F(10,140) = 534.83, *p* < 0.001]. Bonferroni-corrected pairwise comparisons indicated that tumor volume in the SSD-treated group became significantly reduced compared to the control group from Day 7 onwards (*p* = 0.013 at Day 7, and *p* < 0.001 at each subsequent time point). At Day 21, the relative tumor proliferation rate (T/C %) was 55.77%, and the tumor inhibition rate reached 44.23% based on relative tumor volume (RTV) calculations. The final tumor weight was significantly reduced in the SSD-treated group compared to control (0.714 ± 0.044 g vs. 1.346 ± 0.039 g, *p* < 0.001, Student’s *t*-test), representing a 46.98% inhibition rate (Figure 1B,C). Analysis of body weight using two-way repeated measures ANOVA showed no significant main effect of treatment (F(1,14) = 0.58, *p* = 0.449) and no significant treatment × time interaction (F(10,140) = 1.32, *p* = 0.221), indicating that SSD administration at 5 mg/kg did not significantly affect animal body weight over the 21-day treatment period (Figure 2A,B). The final body weight did not differ significantly between groups (Control: 21.84 ± 0.55 g vs. SSD: 21.74 ± 0.59 g, *p* = 0.731, Student’s *t*-test; Figure 2B).

In addition, our results of morphological changes in tumor histology showed that the tumor tissue in the untreated group had a compact texture (Figure 1D), an intact capsule, without any neovascularization in the stroma, and almost no apoptotic and necrotic cells were observed. In contrast, the tumor tissue in the SSD-treated group had a looser texture, an intact capsule, with many apoptotic cells and a few necrotic zones, and limited inflammatory cells. Histomorphometric analysis of H&E sections revealed that the central necrotic zone accounted for 32.8 ± 4.6% of the total tumor cross-sectional area in the SSD-treated group (*n* = 8), compared to only 2.4 ± 1.1% in the control group. A central necrotic zone appeared, with the necrotic zone accounting for 1/3 of the total area, with inflammatory cells infiltrating the necrotic zone. There was an inflammatory cell infusion within the zone of necrosis. Therefore, these results suggest an association between SSD exposure and inhibitory effects on tumor growth, prompting further investigation into its potential underlying mechanisms.

### 3.2. SSD Treatment Is Associated with DNA Damage and Cell Cycle Alterations in Ishikawa Cells

**Figure 3:**
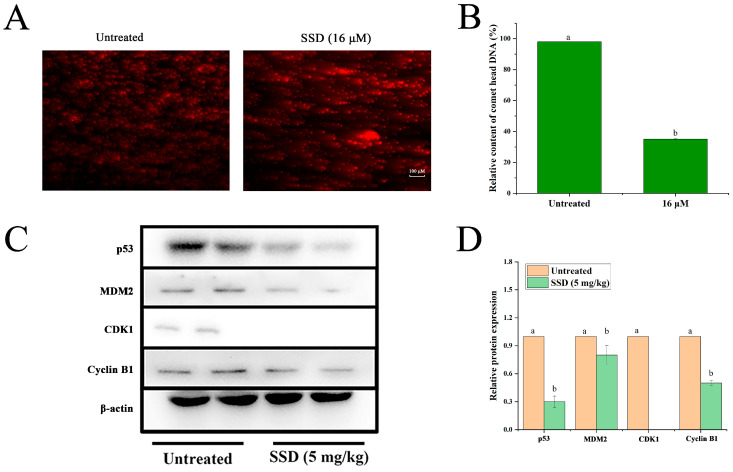
SSD treatment is associated with DNA damage and cell cycle alterations in vivo and in vitro. (**A**) Representative images of the comet assay showing DNA damage. (**B**) Quantitative analysis of DNA damage (mean ± SD, *n* = 3, one-way ANOVA with Tukey’s post hoc test, *p* < 0.05). (**C**) DNA damage- and cell cycle arrest-associated protein bands (*n* = 3). (**D**) Quantitative analysis of DNA damage and cell cycle arrest-related protein expression (mean ± SD, *n* = 3, one-way ANOVA with Tukey’s post hoc test). Lowercase letters indicate significant differences among the groups (*p* < 0.05).

To elucidate mechanisms of SSD-mediated tumor suppression, we first examined effects on DNA integrity and cell cycle progression, as DNA damage-induced checkpoint activation represents a primary anti-tumor mechanism. DNA damage is associated with cell cycle arrest, preventing damaged DNA from continuing to replicate. Single-cell gel electrophoresis (comet assay) revealed that SSD-treated cells exhibited characteristic comet tails indicative of DNA (Figure 3A,B). According to our previous study [25], we found that SSD was associated with G2/M phase arrest in Ishikawa cells and activated the p53-related protein pathway in vitro. To validate these findings in vivo, we analyzed tumor tissue protein expression by Western blotting and found that SSD activated the p53-related protein pathway and downregulated the expression of p53 in vivo (Figure 3C,D). SSD exposure was associated with altered expression of p53-associated pathway proteins in vivo, downregulated the expression of p53, and upregulated the expression of Cyclin B1 and CDK1 proteins.

### 3.3. SSD Treatment Is Associated with Calcium Homeostasis Disruption and ER Stress In Vivo and In Vitro

**Figure 4:**
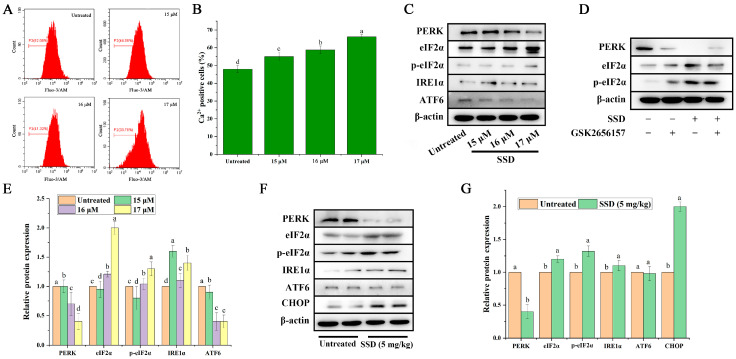
SSD treatment is associated with ER stress phenomena and markers in vivo and in vitro. (**A**) Representative flow cytometry results showing intracellular Ca^2+^ levels. (**B**) Percentage of Ca^2+^ positive cells with increased fluorescence intensity (mean ± SD, *n* = 3, one-way ANOVA with Tukey’s post hoc test). (**C**) Representative ER stress pathway-related protein bands (*n* = 3). (**D**) Representative protein bands of Ishikawa cells after treatment with the PERK inhibitor GSK2656157 (*n* = 3). (**E**) Expression of ER stress pathway-related proteins (mean ± SD, *n* = 3, one-way ANOVA with Tukey’s post hoc test). (**F**) Representative protein bands associated with the ER stress pathway in tumor tissue (*n* = 3). (**G**) Expression of proteins associated with the ER stress pathway in tumor tissue (mean ± SD, *n* = 8 per group, Student’s *t*-test). Different letters indicate statistically significant differences between groups (*p* < 0.05).

Disruption of calcium ion homeostasis was observed as a primary manifestation associated with ER stress following SSD exposure. Fluo-3 AM is one of the most commonly used fluorescent probes for detecting intracellular calcium ion concentration, which can be hydrolyzed by intracellular esterase to form Fluo-3 after entering the cell. Fluo-3 can bind to Ca^2+^, thus generating stronger fluorescence. Flow cytometry analysis demonstrated that SSD treatment was associated with increased Fluo-3 AM fluorescence intensity in a concentration-dependent manner (Figure 4A,B). Meanwhile, Western blot experiments showed that SSD was associated with downregulating the expression of PERK and eIF2α and upregulating the expression of p-eIF2α. To further investigate the association between SSD exposure and Ca^2+^ increase, which affects the expression of endoplasmic reticulum stress-related pathways (Figure 4C–E), we added GSK2656157, an inhibitor of PERK. The downregulation of PERK was significantly affected after treatment with GSK2656157. In addition, we performed Western blotting experiments on mouse tumor tissues, and the results showed that the expression trend of the relevant pathway proteins was consistent between in vivo and in vitro (Figure 4F,G). Importantly, our previous study [25] demonstrated that SSD treatment was significantly associated with upregulation of the Bax/Bcl-2 ratio in a dose-dependent manner, which is consistent with the activation of the intrinsic mitochondrial apoptosis pathway. The present data demonstrate associations between SSD treatment and ER stress markers in the context of calcium homeostasis disruption. However, the direct mechanistic link between these ER stress observations and apoptotic activation was not assessed in the current experimental series and remains to be established. These data indicate that SSD is associated with ER stress markers in vitro and in vivo, consistent with the established literature linking PERK/eIF2α/CHOP signaling to apoptotic pathways, though causality cannot be inferred from the present findings alone.

### 3.4. SSD Treatment Is Associated with Autophagy Induction in Ishikawa Cells In Vivo and In Vitro

**Figure 5:**
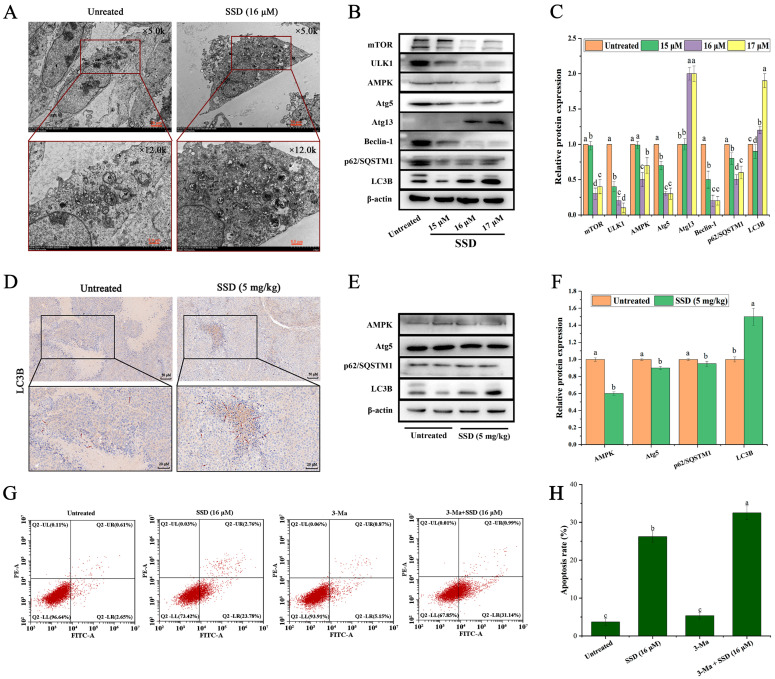
SSD treatment is associated with autophagy markers in vivo and in vitro. (**A**) Representative morphological changes in autophagy in Ishikawa cells visualized by transmission electron microscopy. N, nucleus; M, mitochondria; PS, pseudopods; RER, rough endoplasmic reticulum; Go, Golgi apparatus; ASS, autophagolysosomes; AP, autophagosomes. (**B**) Representative effect of SSD treatment on autophagy-related protein bands in Ishikawa cells (*n* = 3). (**C**) Expression of autophagy-related proteins in Ishikawa cells after SSD treatment (mean ± SD, *n* = 3, one-way ANOVA with Tukey’s post hoc test, *p* < 0.05). (**D**) Representative immunohistochemical analysis of autophagy marker LC3B in mouse tumor tissue sections (*n* = 8 per group). (**E**) Representative protein bands related to the autophagy pathway in mouse tumor tissue (*n* = 3). (**F**) Expression of autophagy pathway-related proteins in mouse tumor tissues (mean ± SD, *n* = 8 per group, Student’s *t*-test). (**G**) Representative flow cytometry analysis of apoptosis in Ishikawa cells treated with SSD (16 μM, 24 h) alone or in combination with the autophagy inhibitor 3-methyladenine (3-Ma, 1 mM). (**H**) Quantitative analysis of the apoptotic rates shown in (**G**) (mean ± SD, *n* = 3, one-way ANOVA with Tukey’s post hoc test, *p* < 0.05). Different letters indicate statistically significant differences between groups (*p* < 0.05).

Parallel to ER stress induction, we examined whether SSD is associated with markers of autophagy, given the interconnection between these stress responses. Autophagy can eliminate damaged organelles and proteins in a self-degradative manner within the cell. The appearance of autophagic vesicles, autophagosomes, and autolysosomes is the main feature of autophagy, and the level of autophagy can be assessed by the LC3-II/I ratio. The results of transmission electron microscopy showed (Figure 5A) that compared with the control, the SSD-treated cells were spindle-shaped, with no obvious disruption of the cell membrane, surrounded by swollen and rounded pseudopods (PS). The cytoplasm was slightly lighter locally, and the number of organelles was abundant; two nuclei (N) were visible, with a uniform distribution of chromatin, and the nuclear membranes were clearly defined; the mitochondria (M) were slightly swollen, with an intact membrane, and the matrix was partially dissolved. And the cristae were fragmented and reduced; rough endoplasmic reticulum (RER) was reduced in number, without obvious expansion and detachment of surface ribosomes; Golgi apparatus (Go) increased in volume, with an increase in the number of cisternae and luminal enlargement; moreover, autophagolysosomes (ASS) and a small number of autophagosomes (AP) were visible.

Western blot analysis revealed an increase in the LC3-II/I ratio associated with SSD exposure and decreased p62/SQSTM1 expression, consistent with an increase in autophagic activity or a blockade in autophagic degradation, suggesting an involvement of the autophagic process (Figure 5B,C). While these protein expression patterns are frequently interpreted as indicators of autophagy induction, we acknowledge that increased LC3-II levels and decreased p62/SQSTM1 expression alone cannot distinguish between enhanced autophagic flux and impaired lysosomal degradation. Without lysosomal inhibitor-based flux assays (e.g., Bafilomycin A1 or Chloroquine), we cannot definitively conclude that SSD induces autophagy with intact flux or that autophagosomes are successfully fusing with lysosomes for degradation rather than accumulating due to lysosomal blockage. The presence of autophagolysosome-like structures (ASS) in TEM images is consistent with autophagy-associated cellular responses, but ultrastructural observations alone cannot establish the functionality or efficiency of the autophagic process. To further explore associations between SSD exposure and markers of autophagy, we performed Western blotting experiments on mouse tumor tissues, and the expression of autophagy-related proteins was consistent with the trend of related proteins in cells (Figure 5E,F). And immunohistochemistry further revealed that the expression of LC3-II was upregulated after SSD treatment (Figure 5D). To determine whether SSD-induced autophagy is associated with a pro-death or pro-survival mechanism, we employed the autophagy inhibitor 3-methyladenine (3-Ma). Pre-treatment with 3-Ma followed by SSD (16 μM) was associated with significantly enhanced apoptosis relative to SSD treatment alone (Figure 5G,H). This finding suggests that SSD-induced autophagy may function as a pro-survival response that potentially attenuates SSD-induced apoptotic cell death. Conversely, pharmacological inhibition of autophagy sensitizes Ishikawa cells to SSD, consistent with a potential combination therapeutic strategy.

### 3.5. SSD Treatment Is Associated with Suppression of Cell Migration and Invasion and Modulation of the MAPK Pathway

**Figure 6:**
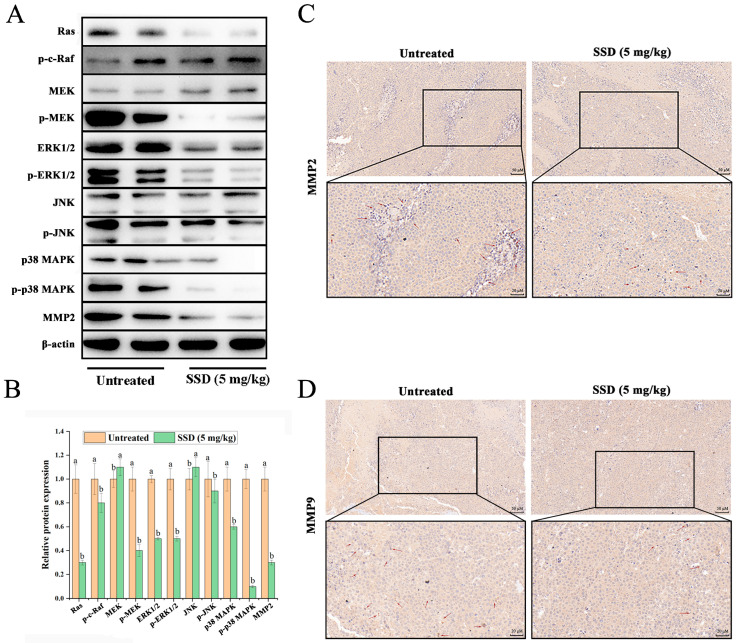
SSD treatment is associated with alterations in the MAPK signaling pathway and cell migration/invasion markers. (**A**) Representative Western blot showing MAPK pathway proteins in mouse tumor tissues. (**B**) Quantitative analysis of protein expression associated with the MAPK pathway-mediated inhibition of cell migration and invasion (mean ± SD, *n* = 3, one-way ANOVA with Tukey’s post hoc test, *p* < 0.05). (**C**,**D**) Representative immunohistochemical analysis of MMP2 and MMP9 proteins in mouse tumor tissues (*n* = 8 per group). Lowercase letters indicate significant differences among the groups within same gene (*p* < 0.05).

Given that metastasis drives cancer mortality, we investigated the effects of SSD on cell migration and invasion, extending our previous in vitro findings to the in vivo context [25]. We examined these mechanisms in an in vivo tumor model. Western blotting analysis of tumor tissues revealed that SSD treatment was associated with downregulation of key proteins involved in cell migration and invasion, including JNK, p38 MAPK, Ras, MMP2, and MMP9 (Figure 6A,B). Immunohistochemical staining confirmed reduced MMP2 and MMP9 expression in SSD-treated tumors (Figure 6C,D). These molecular alterations are consistent with the observed reduction in tumor growth and suggest an association between SSD treatment and suppressed metastatic potential, possibly acting through inhibition of the MAPK-MMP axis.

### 3.6. Effects of SSD on Major Organs of Mice

**Figure 7:**
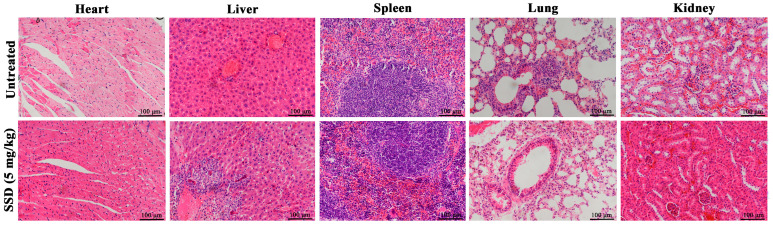
Representative results of H&E staining of heart, liver, spleen, lung and kidney tissues between untreated and treated groups of mice (*n* = 8).

To evaluate the safety of SSD, we assessed the potential toxic effects of SSD on major organ systems. Histopathological examination of heart, liver, spleen, lung, and kidney tissues revealed minimal pathological alterations in SSD-treated mice compared to controls (Figure 7). Specifically, cardiac myocytes maintained normal structure with intact borders and normal myocardium; the interstitium showed no congestion or edema. The splenic capsule remained intact and smooth, with preserved white and red pulp architecture; no significant changes were observed in the number or size of splenic corpuscles in the white pulp, lymphocyte counts were maintained, and minimal congestion was noted in the red pulp. Lung tissue exhibited intact alveolar and bronchial structures without epithelial shedding or luminal secretion; no alveolar or capillary wall thickening, congestion, or edema was observed. Renal glomeruli and tubules appeared structurally preserved without size changes or interstitial congestion. Hepatic lobules maintained normal architecture without hepatocyte necrosis or sinusoidal congestion; however, slight inflammatory cell infiltration was observed in the liver parenchyma.

These findings indicate that SSD at the administered dose predominantly preserves organ integrity, with only minor hepatic inflammatory changes. This observation aligns with our previous finding that SSD exerts minimal cytotoxicity toward normal human embryonic kidney 293 (HEK293) cells [25]. While the slight hepatic infiltration suggests a need for dose optimization in future studies, the overall favorable safety profile supports the continued evaluation of SSD as a potential chemotherapeutic candidate with an acceptable therapeutic window.

## 4. Discussion

Endometrial cancer (EC) is the third most common gynecological malignancy. Despite therapeutic advances, many patients experience poor prognosis and high recurrence rates [33,34], necessitating novel treatment strategies. Natural compounds derived from traditional medicinal plants present an attractive avenue for drug development due to their multi-target potential and favorable safety profiles [35,36]. In our previous study [25], we established that SSD is associated with inhibition of Ishikawa cell proliferation and migration with minimal cytotoxicity to normal HEK293 cells, findings that are consistent with apoptosis markers, G2/M phase accumulation, and reduced migratory capacity. The present study demonstrates associations between SSD administration and reduced tumor volume and weight, without significant alterations in body weight. These findings are consistent with a potential anti-tumor role for SSD. The observed molecular alterations presented below generate hypotheses regarding potential pathways involved.

Cell cycle arrest represents a common cellular response to various stressors, including DNA damage [37]. The comet assay, which detects DNA strand breaks and oxidative damage through the formation of characteristic “comet tail” patterns [38,39], showed that SSD treatment was associated with increased DNA damage in Ishikawa cells. The concurrent observation of G2/M phase accumulation and activation of the p53 signaling pathway provides important clues regarding the nature of DNA damage. The G2/M DNA damage checkpoint is primarily activated by DNA double-strand breaks (DSBs) through the ATM-Chk2-p53-p21 cascade [9]. While these data establish a correlation between SSD exposure, DNA damage, and cell cycle perturbation, causality and the specific lesion type require further investigation using enzyme-modified comet assays and direct DNA damage assessment; neither can we clarify whether these effects represent primary or secondary cellular responses, nor can we clarify the specific type of DNA damage involved. Future studies should employ enzyme-modified comet assays to precisely characterize the DNA lesion spectrum induced by SSD.

The endoplasmic reticulum (ER) serves critical functions in calcium homeostasis and apoptotic signaling [40,41]. The precise temporal and causal relationships between calcium disturbance and ER stress activation represent a critical mechanistic question [40]. In the present study, SSD treatment was associated with elevated intracellular calcium levels and altered expression of ER stress markers, including increased phosphorylation of eIF2α and upregulation of CHOP. Two potential signaling hierarchies may explain these observations. The first is a calcium-centric model where calcium release observed following SSD exposure from ER stores directly activates the PERK pathway via calcium-sensing luminal domains, positioning calcium disturbance as the primary trigger. The second is a membrane-centric model where SSD may directly interact with ER membrane integrity, causing simultaneous calcium leakage and protein misfolding, with calcium release and PERK activation representing parallel downstream consequences [42]. Our data are compatible with both models. The dose-dependent calcium increase preceding PERK activation supports the calcium-centric hierarchy, while the known membrane-active properties of saponins suggest potential direct membrane targeting. Critically, the functional link between ER stress or calcium disturbance and mitochondrial apoptosis execution, though biologically plausible, cannot be definitively established from the present dataset alone. Our previous study [25] reported that SSD significantly upregulates the Bax/Bcl-2 ratio, establishing an association with mitochondrial apoptosis activation; however, those experiments were conducted separately and were not replicated in the current experimental series. The observation that PERK inhibition modulates downstream markers validates the functional importance of the ER stress signaling axis within the current study, but does not establish a mechanistic connection to apoptotic execution in this experimental context. Definitive resolution of whether ER stress directly mediates apoptosis in this system would require concurrent assessment of both pathways within the same experimental series, including evaluation of CHOP-mediated regulation of Bcl-2 family proteins and mitochondrial membrane integrity. Such experiments represent important priorities for future mechanistic studies.

Autophagy represents a complex cellular process involving the formation of autophagosomes and their subsequent fusion with lysosomes to form autolysosomes [43]. Transmission electron microscopy revealed the presence of autophagic vacuoles and autolysosome-like structures in SSD-treated cells. Western blot analysis revealed increased LC3-II/I ratios and decreased expression of autophagy-related proteins, including mTOR, ULK1, p62/SQSTM1, and AMPK. While increased LC3-II levels and decreased p62/SQSTM1 expression are frequently interpreted as indicators of autophagy induction, we acknowledge that these observations alone cannot distinguish between enhanced autophagic flux and impaired lysosomal degradation [44]. Without lysosomal inhibitor-based flux assays (e.g., Bafilomycin A1 or Chloroquine), we cannot definitively conclude that SSD induces autophagy with intact flux. The observed protein expression patterns are consistent with autophagy-associated cellular responses, but the precise nature of these alterations, whether representing induction, blockage, or a combination thereof, remains to be determined through experimental manipulation of autophagic flux.

The functional assessment of autophagy markers observed following SSD exposure using 3-Ma suggests a pro-survival role for this process, wherein autophagy acts as a compensatory mechanism to mitigate SSD-induced cellular stress. This finding has important translational implications: rather than targeting autophagy as a therapeutic endpoint, our data suggest that autophagy inhibition could serve as a sensitization strategy to enhance SSD efficacy. Clinically available autophagy inhibitors such as hydroxychloroquine could be explored in combination with SSD analogs to achieve enhanced anti-tumor effects at reduced doses, potentially improving the therapeutic window.

Tumor cell migration and invasion involve complex signaling networks, including matrix metalloproteinase activity and MAPK pathway signaling [45,46]. Our previous in vitro study [25] demonstrated an association between SSD treatment and reduced cell migration and invasion via MAPK pathway modulation. In the present in vivo study, SSD treatment was associated with decreased expression of MAPK family proteins (including JNK, p38 MAPK, and components of the Ras/Raf/MEK/ERK cascade) as well as reduced MMP2 and MMP9 levels. Immunohistochemical analysis confirmed reduced MMP2 and MMP9 expression in treated tumor tissues. These data establish an association between SSD administration and alterations in migration-related protein expression, though it cannot be determined whether these molecular changes preceded, accompanied, or followed the observed reduction in tumor growth, or whether they represent independent epiphenomena rather than mechanistic mediators.

**Figure 8:**
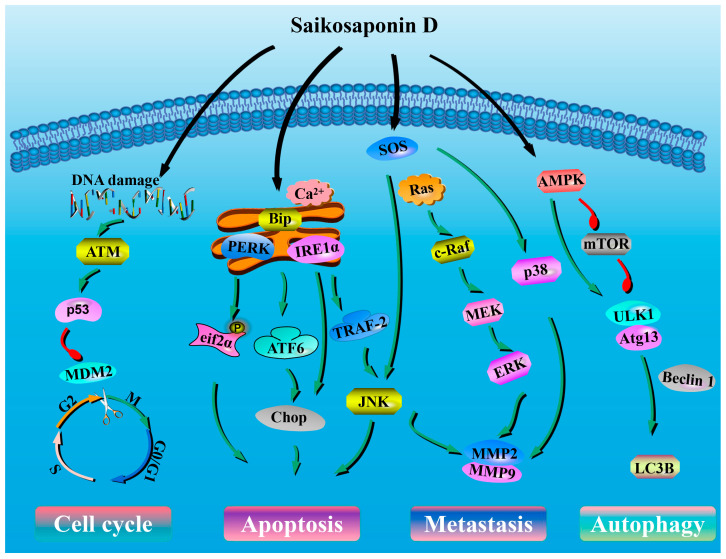
Hypothetical model illustrating potential mechanistic associations between SSD treatment and observed cellular alterations in Ishikawa cells.

The current study has several methodological limitations that need to be acknowledged. While we have demonstrated using 3-Ma that SSD-induced autophagy functions as a pro-survival mechanism, our study did not assess autophagic flux using lysosomal inhibitors such as Bafilomycin A1 or Chloroquine. Such experiments would provide additional confirmation that the observed LC3-II accumulation and p62/SQSTM1 degradation represent intact autophagic flux rather than lysosomal blockage. Future studies should incorporate comprehensive flux assays to further validate these findings. Regarding the relationship between calcium disturbance and ER stress activation, while we have established clear associations between these events, the precise signaling hierarchy remains to be definitively resolved. Specifically, experiments utilizing calcium chelators to determine whether calcium release is the primary trigger or downstream consequence of ER membrane disruption were not performed. Similarly, direct assessment of ER membrane integrity upon SSD exposure would clarify whether membrane targeting represents the initiating event. These mechanistic uncertainties do not diminish the functional importance of the observed PERK activation and apoptotic responses but highlight priorities for future investigation. Tumor volume measurements obtained with calipers are subject to operator-dependent variability. Western blotting provides semi-quantitative protein expression data with potential variability in antibody specificity and loading consistency. Mitochondrial apoptosis assessment was incomplete in the present studies; although previous findings [25] reported Bax/Bcl-2 alterations, their absence here limits integration with ER stress observations. Histological assessments relied on qualitative and semi-quantitative histomorphometric analysis. Despite randomization, residual confounding may arise from unmeasured factors such as variations in cell injection, animal metabolism, culture conditions, or microbiome differences. Our findings are derived from a single-cell line and animal model, limiting generalizability. Additionally, the pharmacokinetics, optimal dosing, and long-term safety of SSD remain to be characterized. Therefore, whether the observed molecular changes precede, accompany, or follow tumor growth inhibition, or represent mechanistic mediators versus epiphenomena, cannot be determined. Altogether, these limitations indicate that the present data demonstrate associations rather than definitive causal mechanisms. Figure 8 provides a schematic summary of the potential mechanistic associations between SSD treatment and the observed cellular alterations. However, we emphasize that these observations demonstrate associations rather than causative mechanisms.

## 5. Conclusions

The present study demonstrates associations between SSD administration in a nude mouse xenograft model of endometrial cancer and reduced tumor growth, as evidenced by decreased tumor volume and weight. These anti-tumor associations were accompanied by alterations in multiple protein expression patterns, including markers related to DNA damage response, cell cycle regulation, ER stress, autophagy, and cell migration and invasion. Notably, functional characterization using the autophagy inhibitor 3-MA suggests that SSD-induced autophagy may serve as a protective mechanism, as pharmacological blockade of autophagy significantly enhanced SSD-induced apoptosis, providing a mechanistic rationale for combination therapeutic strategies. Histological analysis revealed increased necrotic and apoptotic areas in treated tumors. Notably, major organ systems (heart, liver, spleen, lung, and kidney) showed no significant pathological changes under the conditions tested, consistent with a favorable toxicity profile at the administered dose. These findings suggest that SSD may be a promising candidate therapeutic agent for endometrial cancer, with the translational potential of autophagy inhibitor combination strategies warranting further investigation in expanded preclinical models.

## Data Availability

The original contributions presented in this study are included in the article. Further inquiries can be directed at the corresponding author.
